# Visual Electroencephalography Assessment in the Diagnosis and Prognosis of Cognitive Disorders

**DOI:** 10.1097/WNP.0000000000001107

**Published:** 2024-07-25

**Authors:** Daan M. Michels, Sjoerd van Marum, Samuel Arends, D. L. J. Tavy, Paul W. Wirtz, Bas S. F. T. M. de Bruijn

**Affiliations:** * Department of Neurology and Clinical Neurophysiology, Haga Hospital, The Hague, the Netherlands;; ‡ Medical Faculty, LUMC, Leiden, the Netherlands; and; † Department of Neurology, Erasmus MC, Rotterdam, the Netherlands.

**Keywords:** Electroencephalography, Dementia, Alzheimer disease, Vascular dementia, Frontotemporal dementia, Dementia with Lewy bodies, Mild cognitive impairment, Intermittent photic stimulation

## Abstract

**Purpose:**

Electroencephalography (EEG) is a noninvasive diagnostic tool that can be of diagnostic value in patients with cognitive disorders. In recent years, increasing emphasis has been on quantitative EEG analysis, which is not easily accessible in clinical practice. The aim of this study was to assess the diagnostic and prognostic value of visual EEG assessment to distinguish different causes of cognitive disorders.

**Methods:**

Patients with cognitive disorders from a specialized memory clinic cohort underwent routine workup including EEG, neuropsychological testing and brain imaging. Electroencephalography parameters including posterior dominant rhythm, background activity, and response to photic stimulation (intermittent photic stimulation) were visually scored. Final diagnosis was made by an expert panel.

**Results:**

A total of 501 patients were included and underwent full diagnostic workup. One hundred eighty-three patients had dementia (111 Alzheimer disease, 30 vascular dementia, 15 frontotemporal dementia, and 9 dementia with Lewy bodies), 66 patients were classified as mild cognitive impairment, and in 176, no neurologic diagnosis was made. Electroencephalography was abnormal in 60% to 90% of patients with mild cognitive impairment and dementia, most profoundly in dementia with Lewy bodies and Alzheimer disease, while frontotemporal dementia had normal EEG relatively often. Only 30% of those without neurologic diagnosis had EEG abnormalities, mainly a diminished intermittent photic stimulation response. Odds ratio of conversion to dementia was 6.1 [1.5–24.7] for patients with mild cognitive impairment with abnormal background activity, compared with those with normal EEG.

**Conclusions:**

Visual EEG assessment has diagnostic and prognostic value in clinical practice to distinguish patients with memory complaints without underlying neurologic disorder from patients with mild cognitive impairment or dementia.

Dementia is a global health care problem of increasing magnitude. In 2019, an estimated 57 million people worldwide were living with dementia, and this number will likely increase to 150 million by 2050.^1^ Alzheimer disease (AD) is the most prevalent form of dementia (70% of all cases), followed by vascular dementia (VD) and dementia with Lewy Bodies (DLB) both accounting for 5% to 10%.^2^

Neuropsychiatric symptoms and cognitive disorders (e.g., disorders of executive functions, information processing, and verbal memory) are frequently present in both dementia and primary psychiatric diseases.^3–5^ Distinction between early stage dementia and other causes of cognitive disorders (such as depression or temporal lobe epilepsy) can be difficult in patients with mild cognitive impairment (MCI). This highlights the need for additional biomarkers to facilitate early patient selection for trials and potential new treatment options.

Electroencephalography (EEG) is a widely accessible, inexpensive, and noninvasive diagnostic instrument. The diagnostic value have been investigated extensively in a variety of neurodegenerative disorders, such as AD, Parkinson disease, and DLB.^6,7^ Electroencephalography can be of additional value in distinguishing cases of DLB and AD from healthy controls.^8,9^ Especially in DLB, EEG abnormalities such as slowing of posterior dominant rhythm (PDR) and presence of frontal intermittent rhythmic delta activity can support the diagnosis in the prodromal phase of disease, when only mild cognitive complaints are present.^10^ In DLB diagnostic criteria, the presence of frontal intermittent rhythmic delta activity has been incorporated as a supportive biomarker.^11^

The grand total EEG score, a published visual EEG assessment scale to determine disease severity, was significantly related to diagnosis in patients with AD compared with healthy controls, and in DLB compared with patients with AD.^9,12^

Intermittent photic stimulation (IPS) is a commonly performed procedure as part of standard EEG recording.^13^ Besides the usage of IPS to detect photosensitivity in epilepsies, suppression of the PDR by IPS is a measure of brain reactivity to stimuli. Previous studies have demonstrated reduced reactivity to IPS in patients with AD compared with healthy controls, but little is known about the value of IPS in other forms of dementia, and potential prognostic value related to symptom progression.^14–17^

The aims of this study were (1) to describe the EEG characteristics in a large cohort from our specialized memory clinic; (2) to assess the diagnostic value of visual EEG assessment and the additional value of IPS, in discriminating different causes of cognitive disorders; and (3) to determine the ability of IPS to discriminate between patients with MCI with and without progression to dementia.

## METHODS

### Study Population

Patients visiting a specialized memory clinic from the neurology department of a large general hospital in The Hague, The Netherlands, were consecutively included from visits between 2016 and 2021. All patients showing deficits on bedside cognitive screening including mini mental state examination, Montreal cognitive assessment, and/or frontal assessment battery were included and underwent diagnostic workup. The diagnostic workup consisted of standardized neuropsychological tests, structural brain imaging (MRI when possible, otherwise computed tomography), laboratory testing (blood cell count; renal, liver, and thyroid function; and vitamins and glucose levels), and a resting state EEG. Additional tests were performed based on clinical indication in individual cases, including nuclear imaging (Fluorodeoxyglucose, amyloid or 18F-N-(3-fluoropropyl)-2β-carboxymethoxy-3β-(4-iodophenyl) nortropane-positron emission tomography (FDG-PET, amyloid-PET, or FP-CIT)) or cerebrospinal fluid analysis (Aβ-42, tau and p-tau).

Final diagnosis was made by an expert panel consisting of multiple neurologists specializing in cognitive disorders, a neuropsychologist, radiologist, and clinical neurophysiologist. All diagnoses of neurodegenerative dementia were based on current international diagnostic criteria.^11^ Electroencephalography was routinely performed to rule out temporal lobe epilepsy as a potential cause of cognitive disorders. The EEG was not part of the diagnostic criteria used, except for the minor role of supportive criterion in DLB.

Mild cognitive impairment was defined as cognitive disorders on one or more cognitive domains, but not fulfilling the diagnostic criteria for dementia (i.e., no interference with activities of daily living). For the purpose of this study, cognitive disorders due to other causes, such as epilepsy, psychiatric disorders, or traumatic brain injury, were not labelled MCI but according to the suspected underlying cause.

### Protocol Approval and Consent

The study was approved by the regional medical ethics committee of the Haga hospital.

### EEG Recording

All participants underwent a 20-minute resting-state EEG (BrainRT version 4.04, O.S.G BV, Belgium). A total of 23 scalp electrodes were placed according to the international 10 to 20 system at the following locations: FP2, FP1, F8, F7, F4, F3, PG2, PG1, A2, A1, T4, T3, C4, C3, T6, T5, P4, P3, O2, O1, Fz, Cz, and Pz. High-frequency and low-frequency pass filters were set at 70 and 0.27 Hz, respectively. Standard recording protocol included alternating between eyes open and closed, hyperventilation during 3 minutes, and IPS. Intermittent photic stimulation was performed according to international standards,^13^ using 4 to 30 Hz IPS frequencies. Patients were seated upright in a slightly darkened room with eyes open. The same flash stimulator and setup was used throughout the entire study period.

### EEG Assessment

Electroencephalographies were scored based on consensus by two reviewers. To determine interobserver agreement, a third clinical neurophysiologist reviewed a subset of EEGs. Assessment was performed both visually and quantitatively, using power spectral density measurement.

Posterior dominant rhythm and screening for diffuse slow background activity (BA) were assessed in at least three artefact-free epochs of sufficient length (at least 5 seconds) using a bipolar montage (double banana). Posterior dominant rhythm was defined as the dominant rhythmic activity over the posterior regions and scored for frequency (Hz) and reactivity (present/absent). Background activity was scored on a four-point scale based on the grand total EEG score previously described by Jonkman et al.^18^: 0 = normal, 1 = intermittent theta activity, 2 = profound theta and some delta activity, and 3 = profound delta activity. The presence of frontal intermittent rhythmic delta activity was scored (present/absent). Focal abnormalities and reaction to IPS were scored using a common average reference or source derivation montage. Abnormal IPS response was defined as the absence of a distinguishable frequency peak on power spectral density, corresponding to the frequency presented with IPS, in at least half of all presented frequencies. Reactivity at subharmonic or supraharmonic frequencies was deemed a physiological variant. A diminished response was scored when a frequency peak was visible, but with power than the BA (in µV^2^/Hz).

### Statistical Analysis

IBM SPSS statistics, version 28, was used for statistical analysis. Clinical and EEG parameters were assessed with χ^2^ test or Kruskal–Wallis test for categorical data. Ordinal and (semi-)continuous data were analyzed with analysis of variance for normally distributed data. A *P*-value of <0.05 was considered statistically significant.

Cohen κ was calculated to determine interobserver agreement.

A multivariate logistic regression model was constructed to determine the prognostic value of EEG parameters for final diagnosis, and likelihood ratios were calculated to distinguish between dementia syndromes and nonneurologic disorders.

## RESULTS

### Patient Characteristics

Between 2016 and 2022, a total of 1,369 patients visited the outpatient clinic for cognitive complaints. After screening, a total of 506 patients received a full diagnostic workup. Of these, five patients were excluded because the EEG data could not be retrieved. Finally, 501 patients were included in this study. The baseline characteristics are summarized in Table 1.

**TABLE 1.** Patient Characteristics of the Overall Population and of Important Subgroups

	Total (*N* = 501)	No Neurologic Disorder (*N* = 176)	MCI (*N* = 66)	AD (*N* = 111)	VD (*N* = 30)	AD/VD (*N* = 18)	FTD (*N* = 15)	DLB (*N* = 9)
Age, years (SD)	67 (11.8)	60 (11.9)	71 (7.8)	73 (8.0)	71 (7.2)	75 (4.9)	71 (6.9)	70 (7.3)
Sex (%)								
Male	281 (56.1)	94 (53.4)	38 (57.6)	49 (44.1)	22 (73.3)	8 (44.4)	9 (57.1)	7 (77.8)
Female	220 (43.9)	82 (46.6)	28 (42.4)	62 (55.9)	8 (26.7)	10 (55.6)	6 (42.9)	2 (22.2)
MMSE (SD, IQR)	25 (4.1, 22–28)	27 (4.1, 25–29)	25 (3.0, 23–26)	23 (4.2, 20–26)	25 (5.0, 22–28)	24 (3.7, 20–26)	25 (2.7, 23–28)	24 (2.6, 23–27)
MoCA (SD, IQR)	21 (5.1, 17–24)	23 (5.4, 20–27)	22 (3.8, 20–24)	18 (3.4, 16–20)	21 (2.7, 20–24)	15 (4.0, 11–17)	22 (7.1, N/A)	16 (7.1, N/A)
FAB (SD, IQR)	14 (3.4, 11–16)	15 (3.2, 13–17)	14 (3.6, 11–16)	12 (3.4, 9–14)	13 (4.0, 10–14)	12 (2.7, 10–14)	14 (2.4, 12–15)	11 (3.0, 8–13)
Current substance abuse								
Alcohol (%)	49 (9.8)	9 (5.1)	11 (16.7)	7 (6.3)	6 (20.0)	2 (11.1)	1 (6.7)	—
Drugs (%)	7 (1.4)	3 (1.7)	1 (1.5)	—	—	—	—	—
Medication affecting EEG (%)								
Benzodiazepines	33 (6.6)	11 (6.3)	5 (7.6)	6 (5.4)	1 (3.3)	2 (11.1)	—	1 (11.1)
Antiseizure medication	26 (5.2)	9 (5.1)	4 (6.1)	1 (0.9)	2 (6.7)	1 (5.6)	—	1 (11.1)
Antipsychotics	7 (1.4)	4 (2.3)	1 (1.5)	1 (0.9)	—	—	1 (6.7)	—
Antidepressants	50 (10.0)	31 (17.6)	2 (3.0)	9 (8.1)	—	2 (11.1)	2 (13.3)	—
EEG characteristics								
PDR peak frequency, Hz (SD, IQR)	9.3 (1.3, 8.5–10.0)	9.8 (1.2, 9.0–10.5)	9.1 (1.2, 8.3–10.0)	8.8 (1.4, 7.8–10.0)	8.9 (0.9, 8.4–9.5)	8.4 (1.2, 7.6–9.4)	9.7 (0.9, 9.1–10.4)	7.6 (1.1, 6.7–8.7)
Background activity (%)								
Normal	339 (67.7)	154 (87.5)	40 (60.6)	53 (47.7)	18 (60.0)	6 (33.3)	11 (73.3)	1 (11.1)
Mild slowing	121 (24.2)	20 (11.4)	22 (33.3)	42 (37.8)	8 (26.7)	9 (50)	4 (26.7)	4 (44.4)
Moderate slowing	34 (6.6)	1 (0.6)	4 (6.1)	15 (13.5)	3 (10)	3 (16.7)	—	3 (33.3)
Severe slowing/absent	4 (0.8)	1 (0.6)	—	—	—	—	—	1 (11.1)
FIRDA (%)	56 (11.2)	8 (4.5)	6 (9.1)	22 (19.8)	4 (13.3)	3 (16.7)	1 (7.1)	6 (66.7)
Epileptiform discharges (%)	36 (7.2)	12 (6.8)	2 (4.4)	6 (5.4)	2 (6.7)	1 (5.6)	—	—
Generalized	3 (0.6)	1 (0.6)	1 (1.5)	1 (0.9)				
Focal								
Frontal	24 (5.4)	6 (3.4)	—	5 (4.5)	—	1 (5.6)	1 (6.7)	—
Temporal	32 (6.4)	9 (5.1)	1 (1.5)	5 (4.5)	2 (6.7)	—	1 (6.7)	—
IPS reactivity (%)								
Normal	298 (59.5)	124 (70.5)	32 (48.5)	60 (54.1)	17 (56.7)	9 (50.0)	3 (33.3%)	8 (53.3)
Diminished	115 (23.0)	31 (17.6)	23 (34.8)	29 (26.1)	6 (20.0)	5 (27.8)	—	2 (13.3)
Absent	79 (15.8)	16 (9.1)	9 (13.6)	22 (19.8)	7 (23.3)	4 (22.2)	6 (66.7%)	5 (33.3)

Percentages may not add up to 100% due to the exclusion of minor subgroups from this table.

AD, Alzheimer disease; DLB, dementia with Lewy bodies; EEG, electroencephalography; FAB, frontal assessment battery; FIRDA, frontal intermittent rhythmic delta activity; FTD, frontotemporal dementia; IPS, intermittent photic stimulation; IQR, interquartile range; MCI, mild cognitive impairment; MMSE, mini mental-state examination; MoCA, montreal cognitive assessment; PDR, posterior dominant rhythm; VD, vascular dementia.

A total of 183 patients fulfilled the clinical criteria for dementia, specifically AD in 111 patients (22.2% of the total population), VD in 30 patients (6%), a combination of AD and VD in 18 cases (3.6%), frontotemporal dementia (FTD) in 15 cases (3%), and DLB in 9 (1.8%). In total, 66 patients (13.2%) had a possible neurodegenerative disorder but did not fulfil the criteria for dementia and were therefore classified as MCI. Patients without evidence of a neurologic condition were significantly younger than both patients with MCI and dementia (mean age 60 [SD 11.9] vs. 72 [SD 7.7] years, *P* < 0.001).

Other causes of cognitive disorders were toxic encephalopathy due to the (over)use of alcohol, drugs or medication (4%), structural brain abnormalities (3.8%), and temporal lobe epilepsy (1.2%). In 176 patients (35.1%), no underlying condition (neurologic or otherwise) was diagnosed.

### EEG Characteristics

**FIG. 1.**
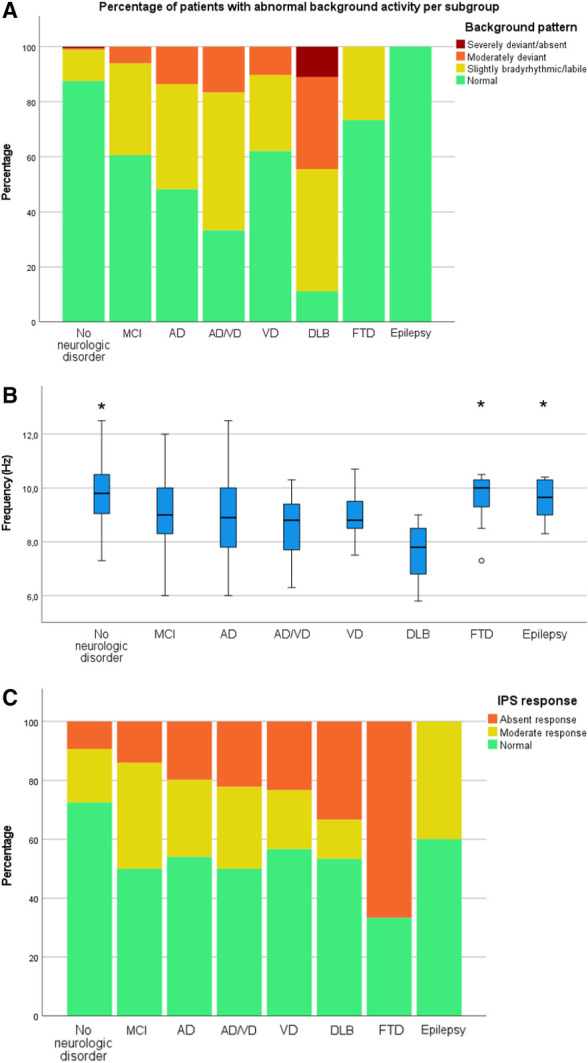
**A**, Percentage of patients with abnormal BA per subgroup. **B**, Posterior dominant rhythm peak frequency per subgroup. *P < 0.05. **C**, Percentage of patients with abnormal IPS response per subgroup. AD, Alzheimer disease; BA, background activity; DLB, dementia with Lewy bodies; FTD, frontotemporal dementia; IPS, intermittent photic stimulation; MCI, mild cognitive impairment; VD, vascular dementia.

Figures 1A–1C show an overview of the BA, PDR, and IPS response for each specified subgroup. Cohen κ was 0.62, indicating substantial interobserver agreement between EEG raters. Background activity was normal in 88% of those without a neurologic condition, compared with 47% of patients with AD, 39% of patients with AD/VD, and 11% of patients with DLB. All epileptic patients had normal BA (Fig. 1A). Posterior dominant rhythm peak frequency was significantly higher for those without neurologic disorder, but also for patients with epilepsy and FTD, compared with other neurodegenerative disorders (Fig. 1B). Aside from FTD, no significant differences in BA and PDR frequency between subgroups of dementia were observed.

Response to IPS either diminished or absent in approximately 50% to 66% of patients with MCI and dementia, while this was only the case for 27% of those with no neurologic disorder. In contrast to BA, which is relatively normal in patients with FTD, IPS response was absent in 67%, compared with around 20% to 33% of patients with AD, AD/VD, and DLB (Fig. 1C).

**FIG. 2.**
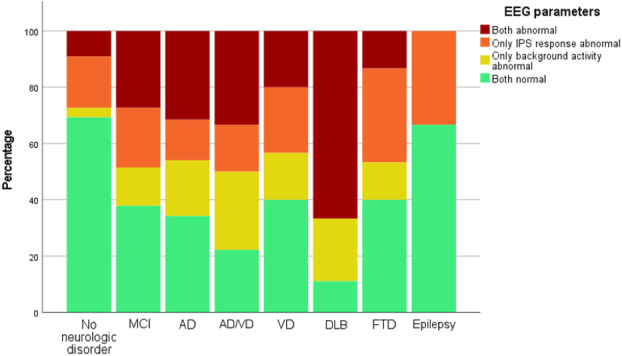
Percentage of patients with any EEG abnormality per subgroup. AD, Alzheimer disease; DLB, dementia with Lewy bodies; EEG, electroencephalography; FTD, frontotemporal dementia; IPS, intermittent photic simulation; MCI, mild cognitive impairment; VD, vascular dementia.

When BA and IPS response were combined, patients with neurodegenerative disease had more abnormalities than those with no neurologic disorder or epilepsy (60% to 65% vs. 35% to 40%, respectively). The most EEG abnormalities (80% to 90%) were seen in patients with combined AD/VD and DLB (Fig. 2).

### Diagnostic Classification Based on EEG

The predictive value of different EEG parameters was assessed using a backward logistic regression model, corrected for age and sex. Odds ratios for a diagnosis of MCI and AD, compared with no neurologic disorder, are summarized in Table 2. Abnormal BA (OR 3.7 [95% CI: 2.2–6.2], *P* < 0.001), presence of frontal intermittent rhythmic delta activity (OR 4.4 [95% CI: 1.7–11.2], *P* = 0.002), and abnormal response to IPS were all independent predictors of AD. For MCI, only abnormal BA (OR 2.2 [95% CI: 1.2–3.9], *P* = 0.008) was a statistically significant predictor.

**TABLE 2.** Relationship Between EEG Parameters and Clinical Diagnosis of MCI (*n* = 66) or AD (*N* = 128) Compared With No Neurologic Disorder (*n* = 176), Based on Backward Logistic Regression, Adjusted for Age and Sex

EEG Parameter	OR for MCI	95% CI	*P*	OR for AD	95% CI	*P*
Abnormal background activity	2.2	1.2–3.9	0.008	3.7	2.2–6.2	<0.001
Presence of FIRDA	1.7	0.5–5.3	0.39	4.4	1.7–11.2	0.002
Abnormal response to IPS	1.4	0.9–2.2	0.134	1.5	1.0–2.2	0.038
Any epileptic activity	2.5	0.5–12.0	0.26	1.3	0.4–3.8	0.67

AD, Alzheimer disease; CI, confidence interval; EEG, electroencephalography; FIRDA, frontal intermittent rhythmic delta activity; MCI, mild cognitive impairment; OR, odds ratio.

The diagnostic value of abnormal BA for AD and “any dementia” was calculated based on 2 × 2 tables, comparing with those without neurologic disorders (Tables 3 and 4). Sensitivity and specificity were 0.52 and 0.88 for AD, and 0.51 and 0.88 for “any dementia.” These result in positive likelihood ratios of 4.15 (95% CI: 2.70–6.38) (AD) and 4.02 (95% CI: 2.68–6.17) (any dementia), when abnormal BA is present. Negative likelihood ratios were 0.55 (95% CI: 0.45–0.67) and 0.56 (95% CI: 0.48–0.66), respectively.

**TABLE 3.** Test Characteristics of Abnormal Background Activity for Diagnosing AD

	AD	Non Neurologic Disorder	Total
Background activity abnormal	57	22	79
Background activity normal	53	154	207
Total	110	176	286

Sensitivity: 0.52. Specificity 0.88. Positive likelihood ratio: 4.15 (95% CI: 2.70–6.38). Negative likelihood ratio: 0.55 (95% CI: 0.45–0.67).

AD, Alzheimer disease.

**TABLE 4.** Test Characteristics of Abnormal Background Activity for Diagnosing Any Dementia*

	Any Dementia*	Non Neurologic Disorder	Total
Background activity abnormal	91	22	113
Background activity normal	90	154	244
Total	181	176	357

Sensitivity: 0.51. Specificity 0.88. Positive likelihood ratio: 4.07 (95% CI: 2.68–6.17). Negative likelihood ratio: 0.56 (95% CI: 0.48–0.66).

* Alzheimer disease, vascular dementia, dementia with Lewy bodies, or frontotemporal dementia.

### EEG-Based Prediction of Conversion to Dementia for Patients with MCI

**FIG. 3.**
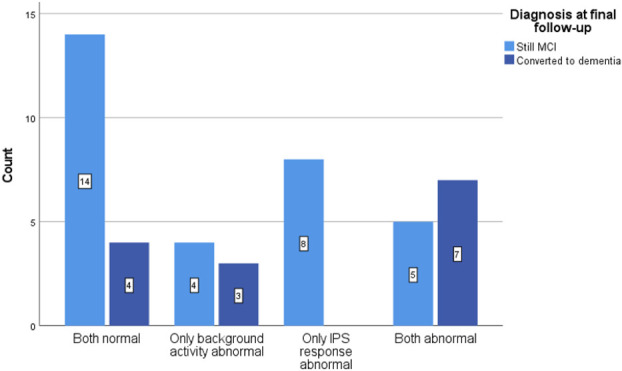
Amount of patients converted from MCI to dementia based on EEG abnormalities at follow-up (median 20 months). OR for progression to dementia in case of abnormal BA: 6.1 (95% CI: 1.5–24.7). OR for progression to dementia in case of abnormal response to IPS: OR 1.4 (95% CI: 0.4–4.9). BA, background activity; EEG, electroencephalography; IPS, intermittent photic stimulation; MCI, mild cognitive impairment; OR, odds ratio.

Of the 66 patients initially diagnosed with MCI, 45 had a clinical follow-up (median duration 20 months [IQR: 14–33 months]). During this period, 14 patients demonstrated progression of symptoms and fulfilled the criteria for clinical dementia (10 AD, 1 VD, 1 combined AD/VD, and 2 DLB). The EEG characteristics of all MCI cases with clinical follow-up are shown in Fig. 3. Those with EEG abnormalities at baseline had a higher chance of converting to dementia compared with those with normal EEG. However, only abnormal BA significantly increased the risk of progression to dementia for patients with MCI (OR 6.1 [95% CI: 1.5–24.7]), whereas abnormal response to IPS in the absence of other EEG abnormalities did not significantly increase the risk of conversion (OR 1.4 [95% CI: 0.4–4.9]). The likelihood of conversion to dementia was 2.46 (95% CI: 1.29–4.68) for abnormal BA versus 0.56 (95% CI: 0.48–0.66) for normal BA (Table 5).

**TABLE 5.** Likelihood of Conversion From MCI to Dementia Based on Abnormal Background Activity at Clinical Follow-Up (Median Duration 20 months)

	Conversion To Dementia	Still MCI	Total
Background activity abnormal	10	9	19
Background activity normal	4	22	26
Total	14	31	45

Sensitivity: 0.71. Specificity 0.71. Positive likelihood ratio: 2.46 (95% CI: 1.29–4.68). Negative likelihood ratio: 0.40 (95% CI: 0.17–0.95).

MCI, mild cognitive impairment.

## DISCUSSION

The aims of this study were (1) to describe the EEG characteristics in a large cohort from our specialized memory clinic; (2) to assess the diagnostic value of visual EEG assessment and the additional value of IPS, in discriminating different causes of cognitive disorders; and (3) to determine the ability of IPS to discriminate between patients with MCI with and without progression to dementia.

Our results show that visual assessment of resting-state EEG has diagnostic value in patients with cognitive complaints. Abnormal slowing of BA and decreased PDR peak frequency were the most reliable parameters to distinguish patients with neurodegenerative disease from those with epilepsy or without any neurologic diagnosis. Background activity was normal in 88% of patients without a neurologic disorder, and only 11% to 60% of those with various types of dementia or MCI. Visual interpretation of EEG in the setting of cognitive disorders evaluates the nature of rhythmic BA and the presence of (paroxysmal) slow activity.^12,19^ The use of EEG in patients with cognitive disorders has been extensively researched, and a similar relationship has previously been demonstrated between increased slow activity on EEG and performance on cognitive tests.^20^ Several studies have found that EEG patterns differ depending on the underlying cause of cognitive complaints.^6,7,9,21^ Especially, patients with DLB are known to demonstrate profound EEG abnormalities, irrespective of their age, when compared with patients with AD.^8,21^ Patients with FTD in our study generally had normal BA and PDR. These findings match those from previous studies, which found EEG to be less affected in patients with FTD compared with AD cases, despite marked cognitive deficits.^22^

We also studied the added diagnostic value of response to IPS to distinguish severity and different causes of cognitive disorders. Some studies have demonstrated reduced responsiveness in patients with AD compared with healthy controls, especially for higher stimulation frequencies.^14–17,23^

We observed abnormal IPS response in around 46% to 67% of those with MCI or dementia, compared with 30% of participants without a neurologic diagnosis. These differences were smaller than expected based on previous studies and not statistically significant. This might be explained by technical and procedural aspects of IPS testing, which requires cooperation and the sustained attention of the subject.^13,24^ Although participants may not have had a neurologic cause of their symptoms, they still suffered from cognitive disorders that could have affected their attention levels and the ability to retain focus during IPS, especially in a dim-lit room. In contrast to our study population, many previous studies compared patients with AD with healthy controls without cognitive complaints, who may have had better attention span. Most studies also had relatively small sample sizes. This may explain why in our study abnormal IPS response was not an independent predictor of MCI and only a minor predictor of AD compared with patients without neurologic disorders (Table 6).

**TABLE 6.** Correlations Between EEG Parameters and Other Diagnostic Tests in Patients With Memory Complaints

	Background Activity		IPS Response	
	Spearman ρ	*P*	Spearman ρ	*P*
Imaging				
GCA scale	0.36	<0.001	0.30	<0.001
MTA score	0.34	<0.001	0.28	<0.001
Fazekas scale	0.21	<0.001	0.12	0.006
CSF				
Amyloid-42 level	0.30	0.013	0.20	0.1

CSF, cerebrospinal fluid; EEG, electroencephalography; GCA, global cortical atrophy; IPS, intermittent photic stimulation; MTA, medial temporal lobe atrophy.

Third, we studied the prognostic value of EEG in patients with MCI. We found that the presence of abnormal BA on baseline EEG can be used as a prognostic tool to predict the chance of progression to clinical dementia in the future. A diminished or absent response to IPS without accompanying EEG abnormalities had no added value. The prognostic value of EEG in patients with MCI, as demonstrated in this study, is in line with previous research.^10,20^

Our results indicate that at initial presentation, abnormal BA is a more useful predictor than a normal EEG. 83% of those with abnormal BA were diagnosed with any form of dementia or MCI, while only 67% with normal BA had no neurologic disorder. Furthermore, when predicting conversion from MCI to dementia, a normal EEG is a more useful predictor. 83% of those with MCI and normal BA did not convert to dementia during follow-up, whereas 56% of those with abnormal BA did. However, patients who have not yet converted might still convert in the future.

However, prevalence of dementia in our cohort is high, with 37% of participants being diagnosed with “any dementia.” In the general population or general neurologists office, prevalence of dementia will likely be lower. Studies conducted in the emergency department found dementia documented in the medical history of 3% to 6% of presented patients.^25^ Routine bedside screening tests showed signs of dementia in up to 21.5% of patients older than 70 years admitted to the emergency department.^26^ With lower prevalence, the usefulness of a normal test result to rule out dementia increases. It is important to note that, with the exception of FTD, all types of dementia and even MCI show abnormal BA in a significant portion of patients (DLB being the most profound), limiting the ability to distinguish between dementia subgroups based on EEG alone.

There are several strengths to our study. First, we describe a large consecutive cohort comprising of several etiological subgroups of dementia, with a distribution that corresponds well to existing epidemiological data. Furthermore, our reference group consists of actual patients referred for cognitive complaints, but without a neurologic cause for these complaints. This makes our results more applicable to the general population compared with studies that used healthy controls.

Second, all participants underwent a standardized diagnostic workup, and the final diagnosis was established by a multidisciplinary expert panel, limiting the risk of selection or information bias.

Third, the emphasis on visual EEG assessment makes for easy interpretation and facilitates the applicability in clinical practice. In recent years, the main focus of many researchers has been on quantitative EEG analysis, such as random forest classifiers and other machine learning algorithms. These studies have demonstrated a high diagnostic yield for patients with DLB and AD, with sensitivity and specificity values around 90%.^27–29^ However, such techniques are laborsome and require advanced computing skills not possessed by many clinicians. Therefore, they are currently of limited value for the general clinical neurologist. This amplifies the need for visually assessable and reliable EEG parameters in patients with cognitive disorders.

However, we acknowledge the fact that quantitative analysis of EEG data improves diagnostic accuracy, which is lost in visual analysis. This is a limitation of our study. Moreover, although we do have longitudinal data on a significant portion of patients with MCI and dementia, those in whom no neurologic diagnosis was made were generally excluded from follow-up. Some of these patients may have been erroneously classified as nonneurologic, especially if their symptoms were mild. Further studies should therefore focus on long-term follow-up in a larger cohort of patients with mild symptoms to monitor the rate of progression to MCI or dementia. In addition, repeated EEG measurements at standardized intervals could provide more prognostic parameters for the chance of conversion in this group.

In conclusion, this study shows that (1) visual EEG assessment has diagnostic and prognostic value and can distinguish patients with memory complaints without underlying neurologic disorder, from patients with MCI or dementia. (2) Abnormal slowing of BA is the most reliable diagnostic parameter for neurodegenerative disease. Although diminished response to IPS is seen more frequently in patients with MCI and dementia compared with nonneurologic disorders, this finding alone cannot support a specific etiological diagnosis. (3) In patients with MCI, normal EEG is associated with a lower likelihood of progression to dementia. Based on these findings, we suggest the use of standard EEG at an early stage in patients with cognitive complaints in whom uncertainty exists regarding the underlying diagnosis.
